# Caries and Necrosis of the Maxillary Bones

**Published:** 1883-01

**Authors:** Truman W. Brophy

**Affiliations:** Chicago.


### ARTICLE II.

### *Caries and Necrosis of the Maxillary Bones.*

BY TRUMAN W. BROPHY, M. D., D. D. S., CHICAGO.

[Read before Illinois State Dental Society, May, 1882]

Caries and necrosis of bone both result from the removal or death of the periosteum, or from the separation of the periosteum from the bone by means of intervening pus.

These conditions, however, frequently have their origin in ostitis.

The exciting causes of the diseases under consideration are various: confined dentes sapientiæ, for want of room, salivary calculus, traumatism, heavy malleting in filling the teeth of young patients, and other agents capable of producing ostitis or periostitis; but of all the causes of diseases of the maxillary bones, the presence of pulpless teeth and fangs of teeth is far the most prevalent. From such teeth and roots, indeed originate more diseases of the bones in question, than from all the other causes combined. Caries of bone is similar to ulceration of the soft parts, while necrosis may be compared to mortification of gangrene. The chief difference between caries and necrosis, is, that caries progresses slowly, not unlike an ulcer upon the skin; simple caries begins upon the surface of bone and gradually goes deeper and deeper until a considerable portion of bone is destroyed, unless it is arrested either by vital force or surgical interference. On the surface of carious bone small accumulations of pus are formed, which, together with granules of bony substance, find an exit through fistulous openings. The pus emanates from the organic matter of the bone.

When necrosis takes place the preceeding inflammation overwhelms the circulation; there is swelling and intense pain, stasis of the blood, quickly followed by suppuration, the pus makes its exit through fistulous openings or about the necks of the teeth, and the mass of bone thus involved is deprived of its nourishment and consequently its vitality. The sequestrum is surrounded by an inflammatory process, which has the effect of loosening and separating it from the living tissues. In caries the bone is eaten out upon the surface and dissolved; in necrosis it is usually honey-combed throughout the entire circumscribed mass.

The objective signs of caries of the maxillary bones are, usually, not unlike those of a chronic alveolar abscess, with fistulous openings through the bone and gums. Quite frequently, however, the pus discharges through the cheek if the disease be in the superior maxilla, and beneath the chin if in the inferior.

The same degree of irritation exerted in different patients may be followed by different results. In case an irritation of the periosteum be established in a strong, vigorous patient, free from specific taints, it may result in caries of the underlying bone; but necrosis with the exfoliation of a sequestrum is not at all likely to occur. On the other hand, the same degree of irritation, exerted in eases of anæmic, scrofulous, syphilitic, or debilitated patients, might terminate in necrosis and the loss of a large portion or even the whole of the bone affected. The superior maxilla is more often diseased than the inferior. Of the twenty-three cases treated by me during the past year, nineteen were of the superior maxilla, while four were of the inferior.

### DIAGNOSIS.

The diagnosis of caries is usually not attended with difficulty. Through the fistulous openings a probe should be introduced, and carried to the surface of the bone. If caries exists, there will be felt, not only the denuded bone, but a roughened, pitted surface will also be detected, when the instrument is brought in contact with the bone. Caries and necrosis of bone with fistulous openings through the gums adjacent to the bicuspid or molar teeth might be mistaken for abscess of the antrum, if we base our diagnosis upon an ocular examination. In antral abscess, as in caries and necrosis, there is swelling and pain, followed by the discharge of pus into the mouth. By the use of the probe a correct diagnosis can usually be made with ease.

Caries may be complicated with, and in some instances due to antral lesions, but, happily, such instances are of comparatively rare occurrence.

### TREATMENT.

Maxillary caries may be successfully treated by either of the following methods:

1st. All loose roots, or those that can not be made useful by having artificial crowns placed upon them, should be extracted, and all teeth from which caries originates, together with roots that can be crowned, should be treated and filled. If, upon examination, we find that the bone is not diseased to a great extent, if caries has not reached farther than the bone immediately surrounding the apices of the roots of one or two teeth, and has not penetrated deeply into the bone, injections, three or four times a week, of aromatic sulphuric acid, will dissolve the decayed bone and effect a cure. A hypodermic syringe with a long flexible silver point, with blunt end, is an excellent instrument with which to carry the solution through the fistulous opening, and bring it in contact with the affected bone. Before applying the acid the cavity should be thoroughly cleansed and syringed out with a two per cent. solution of carbolic acid, and all pus, loose bone, granules, etc., removed, so that the acid will come in direct contact with the diseased bone and dissolve it. If the affection be in the superior maxilla, the patient should be placed in the recumbent position, the tissues surrounding the fistula should be protected by covering them with napkins of pieces of spunk, and the acid carried into the carious cavity and retained there twenty-five or thirty minutes so as to permit it to act upon the bone. Aromatic sulphuric acid is the dilute sulphuric acid of commerce, a 13½ per cent. solution of $H_2SO$, together with the aromatic parts of cinnamon and ginger. If aromatic sulphuric acid be used undiluted, swelling of the parts is very likely to follow. The first applications, therefore, should be about one part acid to three of water, and gradually increased to full strength, if the case requires it. The causes and diagnostic signs of necrosis of the maxillary bones have been given, in connection with the description of caries. The methods of treatment alone remain to be described.

Patients suffering from periosteal inflammation, which threatens necrosis of the bone, suffer most excruciating pain, and it is at this stage of disease that prompt and vigorous treatment should be employed. Useless roots should be extracted, numerous free and deep incisions should be made over the surface of the bone, saline cathartics should be administered, the patient kept quiet and lying in a half recumbent position, so that gravity will aid in terminating the inflammation, by resolution. If such treatment be resorted to early, the disease may be arrested before the vitality of the osseous tissues is impaired. If, however, we find that necrosis is inevitable, that the sequestrum is forming, the treatment should consist in frequently syringing the parts with carbonized rose water, and attending to the general health of the patient. When the dead bone has separated from the living, incisions should be made so as to enable the operator to seize the sequestrum with forceps and remove it without lacerating the soft parts. After the operation, the cavity should be cleansed, and filled with the crystals of boracic acid. These crystals should be placed in the cavity daily, and in addition to this, if the cavity be large, it should be plugged with cotton, or what is far better, wax. Thus plugging the cavity, and reducing the size of the plug from time to time, so that granulations may form, will prevent a depression of the face, which is certain to result if this precaution is neglected..

Case 1. Mr. A——, aged 45. Very much emaciated, consequent upon the absorption of pus. I was called in consultation with a physician, and upon making an examination, found an opening through the external plate of the superior maxiliary bone just above the first left bicuspid, through which there was a profuse discharge of pus. The first of these openings had been made by a physician for the introduction of a drainage tube. The pulps of both bicuspids had been removed and the canals filled with gold. The fillings in the teeth and roots were removed, and it was ascertained that the upper portion of the canals had been imperfectly filled. From this space gases evidently had made their exit through the apicial foramen, resulting in the formation of alveolar abscesses. A probe introduced into the fistula came in contact with denuded and roughened bone over a space corresponding in size to a 25 cent silver piece. Patient had been suffering from the affection about seven months prior to my seeing him, during which time he had visited a number of physicians, all of whom believed the teeth to be the cause of the trouble; also several dentists, who thought just the opposite, viz.: that the teeth had nothing whatever to do with the condition of affairs. Diagnosis was caries of the bone, originating from alveolar abscesses of the bicuspid teeth. Drainage tubes were inserted to carry off the flow of pus, and treatment was commenced by washing the parts with tepid carbolized water. Next a solution of aromatic sulphuric acid was injected through the fistula and permitted to remain for fifteen or twenty minutes in contact with the bone. In connection constitutional treatment for improving the general health of the patient was employed. Treatment was continued twice each week for a period of four months, at the end of which time suppuration had ceased and granulations filled the cavity, and by the end of the fifth month a complete cure was affected.

Case II. Mr. B——, aged 42. Had previously rendered dental services for patient, and was called in consultation with a physician. Patient had been absent from the city, had taken a severe cold, and had been suffering eight days from a pain which, commencing in the region of the superior incisiors, had rapidly extended back until the bicuspids of either side were involved. When I first saw him he was bordering on septicæmia, his face was very much swollen, eyes nearly closed and cheeks almost on a level with his nose. Pus was oozing from around the necks of all the teeth. Upon opening the mouth a cushion or sack of pus was seen, covering the whole surface of the hard palate, and totally obliterating the palate arch. The gums were pressed downward to such an extent as to cover the ends of the teeth. Upon introducing a probe, denuded bone honeycombed through and through was encountered.

Diagnosis was necrosis of the bones originating in ostitis, and involving all the teeth anterior to the molars.

Treatment consisted in evacuating pus by free incisions over the entire anterior portion of the bone, as well as of the hard palate, and washing with tepid carbolized water. The feeble condition of the patient demanded prompt and effectual treatment. Stimulants, as brandy and quinine, were freely administered. The nurse was instructed to syringe out the cavity four or five times per day with carbolized water. One week subsequent to my first visit, assisted by Prof, Gunn, I removed the affected bones, on both sides anterior to the first molar teeth. Carbolized water was used for washing the wound, tonics were continued, the wound healed quite rapidly, the soft parts contracted, and within three months after the operation an artificial substitute was inserted which almost wholly overcame the deformity.

Case III. Miss G——, age 23, very anæmic and of a scrofulous diathesis, chronic alveolar abscess of the left central and lateral incisors had existed, and were threated in the usual manner by the patient's dentist, but without success. The teeth were finally extracted, but the pus continued to flow as freely as before. The dentist then referred the patient to me, with a letter, explaining the condition in which he found her, the treatment he had employed and the result up to that time. Upon examination, caries of the bone was discovered. Treatment consisted in incisions over the surface, scraping away the softened bone and washing the parts thoroughly with carbolized water. Crystals of boracic acid were placed in the wound and covered with a pledget of cotton. This treatment was continued in connection with tonics and other constitutional remedies for about two months, when a complete cure resulted.

Case IV. A gentleman, 21 years of age, presented himself at my clinic at the dispensary, with a fistulous opening over the left superior central incisor. A probe introduced into this opening could be passed back upon the superior surface of the palatal plate of the left maxilla, about two and one-half inches. The bone along the track of the probe was very much roughened and suppuration was constant and profuse. Diagnosis was caries of the bone. The tooth contained a large amalgam filling upon its disto-appproximal surface. This filling was removed, the root cleansed and filled. The patient was etherized, and an operation consisting of cutting down upon the bone, excising the end of the root, which protruded into the carious cavity, and scraping away the softened bone, was performed. The disease had destroyed nearly all the palatal plate of the bone, together with the alveolar processes surrounding the affected tooth, and posterior to the lateral of the same side, and central of the opposite side. The patient was provided with a syringe, instructed in its use, and discharged. He left the city, and the subsequent history of the case is not known.

Case V. Caries of the inferior maxillary bone emanating from an alveolar abscess of the first left inferior molar.

An amalgam filling in the tooth was removed, and the pulp chamber was found filled with disorganized sero-pus. The chamber and roots were cleansed and filled, and an effort made to effect a cure without an operation, but at the end of two months, there being no marked improvement, I decided to operate. An incision was made through the gums, and engine bur passed in, and the diseased bone cut away. The cavity, which was of considerable size, was cleansed and filled with boracic acid crystals. This treatment was continued from day to day for a period of two weeks, when it was discontinued, and in one month subsequent to operating the patient was well.

Case VI. Mr. W——, aged 47, presented himself at my clinic at Central Free Dispensary, suffering from a severe pain in the side of the inferior maxilla. Pus had burrowed through to the integument, and by backing up, had made its exit opposite the orifice of the duct of steno. The sinus extended below the inferior border of the bone, so low, indeed, that drainage into the mouth was impossible. An external incision was therefore made at the lowest part of the sac. Solutions of carbolic acid were injected into the pus cavity, and the bone, which was diseased by the presence of several fangs of teeth, was, after the removal of these fangs, treated by scraping and removing all carious portions. The usual subsequent treatment was employed, and in three months after the patient first presented himself, further treatment being unnecessary, he was discharged.

Case VII. Mr. K——, aged, 38. Very much emaciated from over work, was directed to me by a neighboring practitioner of dentistry for diagnosis and treatment. Upon examination I found that necrosis of the inferior maxilla, involving the incisor and cuspid teeth, and caused, no doubt, by the large accumulations of salivary calculus upon the teeth, far beneath the margins of the gums, had established itself. Assisted by Prof. Parkes I removed the alveolar processes and superior half of the body of the bone, including the teeth mentioned. The patient made a very rapid recovery. Boracic acid, daily applied to the cavity, and tonics internally, constituted after treatment. The contour of the face was restored by adjusting a plate to fit the space formed by the removal of the bone.

Case VIII. Mr. S——, aged 26. Had, previous to my seeing him, been under treatment by a dentist, with a view to adjusting a gold crown with porcelain face to the root of a left superior central incisor. An abscess had formed, which resisted the usual treatment, and swelling had extended very rapidly, involving the periosteum and overlying parts of the external surface, right superior maxilla. The root was found loosened and the bone surrounding and above it. Pus has burrowed back and elevated the periosteum from the bone as far as the third molar. The patient was etherized, and the necrosed bone, with the root, was removed. Free incisions in several places were made down to the bone, and the patient placed in the hands of another practitioner for further treatment, owing to my being compelled to leave the city.

Case IX. Master L——, aged 13, six years ago had all four permanent molars, which were badly decayed on their masticating surfaces, filled with gold. The mallet was used freely in condensing the fillings ; the patient complained of having suffered intense pain during the operations and for several weeks thereafter, especially in the region of the right superior molar. A fistula formed anterior to the tooth, near the margin of the gums, through which pus had discharged up to the present time, the patient being now under treatment. The carious bone has been removed, the acid treatment is being employed, and improvement is noticable. The exciting cause of the disease in this case was, no doubt, heavy malleting.

*Resume.* To avoid the diseases described, first, always remove loose, valueless roots that cannot be made useful by crowning. Second, always avoid the use of cotton or other material in the filling of roots which will absorb moisture, or admit fluids within the canals, since from these fluids emanate the gases which cause the inflammations that most frequently lead to diseases of the bones hitherto described. Third, never remove a tooth or root, even though the apex is standing up in the carious bony cavity, provided the periosteum intervening the carious cavity and the alveolar border be not dead. This exposed point of the root may be excised, the pulp canal filled, the carious bone removed, and the teeth restored to usefulness.

When chronic alveolar abscesses do not yield readily to the iodine or carbolic acid treatment, caries although perhaps slight, very likely exists, and the sulphuric acid treatment should be resorted to. When patients suffering from acute periostitis of the maxillary bones present themselves to us, we should unhesitatingly, and without delay, make deep and numerous incisions down upon the bone and relieve the engorged blood vessels.—*Transactions of the Illinois State Dental Society.*

